# Ruptured hepatic hydatid cyst with the formation of an abscess and a cutaneous fistula

**DOI:** 10.1590/0037-8682-0497-2022

**Published:** 2022-12-16

**Authors:** Ramazan Orkun Önder, Tumay Bekci, Serdar Aslan

**Affiliations:** 1 Giresun University, Faculty of Medicine, Department of Radiology, Giresun, Turkey.

**FIGURE 1:**
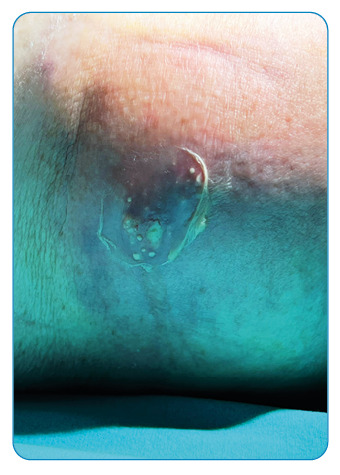
Skin redness and swelling on the right side of the abdomen. Fistulization of the abscess presenting with green abscess content on the skin.

**FIGURE 2:**
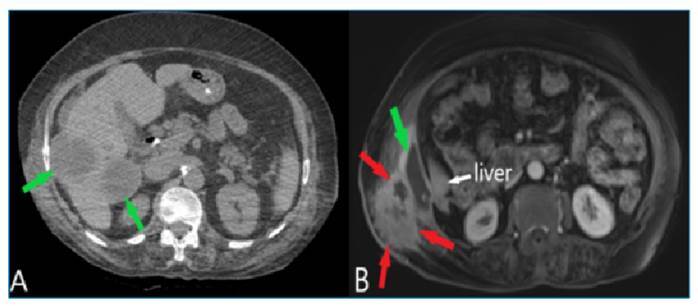
**(A)** The abdominal computed tomography scan performed 1 year before shows HC lesions in the liver (green arrows). **(B)** The MRI scan demonstrates a ruptured HC appearance consistent with a previous liver HC lesion in liver segments VI and VII (green arrow). The gadolinium-enhanced MRI scan shows a peripherally enhanced ruptured HC appearance consistent with abscess formation and accompanying soft tissue infection and fistulization (red arrows).

An 85-year-old woman was admitted to our emergency department with complaints of upper right abdominal quadrant pain, skin redness, and swelling on the right side of the abdomen (Figure 1). The medical record revealed that the patient had been diagnosed with hydatid disease based on computed tomography findings and detected with anti-*Echinococcus* IgG antibodies approximately 1 year before (Figure 2A) and treated with albendazole. Laboratory tests showed elevated C-reactive protein levels (117 mg/L) and white blood cell count (17.7 10⁹/L), and abdominal ultrasonography revealed an unshaped collection in the inferior segment of the right liver lobe spreading to the lateral abdominal wall, with fistulization to the skin. Contrast-enhanced magnetic resonance imaging (MRI) showed a ruptured hydatid cyst (HC) consistent with a previous liver HC lesion in liver segments VI and VII. On gadolinium-enhanced MRI, the ruptured HC appearance was peripherally enhanced, consistent with abscess formation, and accompanying soft tissue infection and fistulization were found (Figure 2B). A drainage catheter was placed in the patient under general anesthesia, and medical treatment was started.

 Cystic echinococcosis is a zoonotic infection that causes >95% of echinococcal diseases in humans^1^. HC rupture is a life-threatening complication that may occur both internally and externally^2^. Abscess formation and the accompanying cutaneous fistulization of HC is an extremely rare complication that may lead to anaphylactic shock and sepsis^3^. Skin changes in the area of previous HC should be alerting, and both clinicians and radiologists should be aware of this extremely rare presentation.
